# Effects of a natural polyphenol on nicotine-induced pancreatic cancer cell proliferation

**DOI:** 10.18332/tid/95159

**Published:** 2018-10-24

**Authors:** Parimal Chowdhury, John J. Jayroe, Bryan E. White, Ember R. Fenton

**Affiliations:** 1 University of Arkansas for Medical Sciences, Little Rock, United States; 2 University of Arkansas at Little Rock (UALR), Little Rock, United States

**Keywords:** resveratrol, MAPK signaling, AR42J cell, oxidative stress

## Abstract

**INTRODUCTION:**

Resveratrol (*trans*-3, 4’, 5-trihydroxystilbene), a phytoalexin derived from the skin of grapes and other fruits, has anti-inflammatory and anti-oxidant effects. Its anti-carcinogenic effects are closely associated with its antioxidant activity; thus, the use of resveratrol as a possible cancer chemo-preventive is considered to be an important area of investigation. In this study we have examined the inhibitory effects of resveratrol in nicotine induced proliferation of pancreatic cancer cells.

**METHODS:**

Cultured AR42J cells were incubated with 100 μM nicotine for 3 min and with 100 μM resveratrol for 30 min, either alone or in combination. Proliferation assays were conducted for a period of 0 to 96 h in serum media, incubated with nicotine and resveratrol, and evaluated by MTT assay. Protein was measured in lysed cells and activation of MAPK signals was measured by western blot using purified p-ERK antibody. Co-localization of activated ERK signals was confirmed by FITC conjugated ERK antibody using immunofluorescence assay and confocal microscopy. Biomarker of lipid peroxidation was determined in cell lysates by malondialdehyde (MDA) bioassay.

**RESULTS:**

Resveratrol significantly suppressed the nicotine-induced proliferation of acinar cells compared to untreated controls (p<0.05). Mitogen activated protein kinase (MAPK) analysis revealed up-regulation of p-ERK expression by nicotine (p<0.05) that was suppressed significantly by resveratrol (p<0.05). Co-localization of activated ERK signals was confirmed by FITC conjugated ERK antibody, and this response was reduced significantly by resveratrol. Nicotine-induced malondialdehyde formation was also suppressed by resveratrol (p<0.05).

**CONCLUSIONS:**

The data suggest that resveratrol suppressed nicotine-induced AR42J cell proliferation. The proliferation of AR42J cells by nicotine is associated with activation of MAPK signals and induction of protein oxidation. Resveratrol suppressed lipid peroxidation and P-ERK activated signals induced by nicotine. We conclude that resveratrol acts as an effective antioxidant in reversing the nicotine induced pancreatic cancer cell proliferation.

## INTRODUCTION

Resveratrol, a naturally occurring phytoalexin and a phenolic compound found in grape skins, mulberries, and certain nuts, is anti-tumorigenic, anti-inflammatory^1-3^ and possess antioxidant properties^1,4^. Resveratrol acts also as a potential cardio-protective agent as it was shown that red wine consumption (a source of resveratrol) is inversely related to cardiovascular disease^5,6^. Besides cardioprotective effects, resveratrol exhibits anticancer activities through a variety of regulatory pathways^7-10^, as documented in several reviews^1,2,11,12^. *In vitro* and *in vivo* studies have confirmed that resveratrol can modulate multiple pathways involved in cell growth, apoptosis, and inflammation^13-15^. Yet, the mechanism aspects of this polyphenol are not completely understood and still under considerable investigation. Jang et al.^3^ reported that chemo-preventative activity of resveratrol may involve three major phases, such as inhibition of cell proliferation, induction of apoptosis, and decreases in cancer progression. Although multiple pathways towards those mechanisms have been investigated^16-18^, the precise mechanism of the anti-proliferative effects of resveratrol, in particular reference to pancreatic cancer, has not been fully evaluated. Golkar et al.^7^ demonstrated that resveratrolinduced growth inhibition in pancreatic cancer cells is mediated in part by up-regulation of macrophage inhibitory cytokine-1 (MIC-1). In human pancreatic cancer cell lines PANC-1 and AsPC-1, resveratrol was shown to inhibit proliferation through apoptosis^8^.

To understand fully the role of resveratrol as an antioxidant/anti-proliferative agent, we have designed this study in a functional rodent immortal pancreatic tumor cell line employing nicotine as an oxidative marker, since it has been shown earlier that nicotine induces increased proliferation of this cell via oxidative mechanisms^19^. Associations between long-term low intensity cigarette smoking and incidence of smoking-related cancer were observed for lifelong smoking of ≤10 cigarettes per day and pancreatic cancer (HR=2.03, 95% CI: 1.05–3.95)^20^, and nicotine may play a major role towards this link. The exact mechanism by which resveratrol acts on pancreatic cancer cells remains unclear to date. In this study, we investigated the role of the MAPK signaling pathway in resveratrol-induced growth inhibition in a rodent pancreatic cancer cell line.

## METHODS

### Cell culture

AR42J cells, a rat pancreatic tumor cell line (ATCC, Rockville, MD) were grown in 75 cm^2^ flasks with 10–12 mL of HAM’s F12 nutrient media with 2 mM L-glutamine and 1.5% NaHCO^3^ (F12K, obtained from Hyclone, Logan, UT), to which 10% fetal bovine serum (FBS) and 1% penicillin-streptomycin were added. The flasks were maintained in the incubator at 37°C, with a 5%-CO2/95%-air atmosphere, until they reached over 80% confluence.

### Cell proliferation studies

These studies were conducted with 100 μM resveratrol, and 100 μM nicotine, treated either alone or in combination with resveratrol followed by exposure to 100 μM of nicotine. A commercially available Cell Viability and Cytotoxicity Assay Kit (Cell Counting Kit, CCK-8, Dojindo Molecular Technologies Inc. Gaithersburg, MD) was used for the assay. Ninety-six well microplates were used and 2 χ 10^4^ cells per well were plated. After attachment for 24 hours in media containing 10% FBS, the cells were maintained in low FBS media overnight before being treated with 100 μM nicotine alone or pretreated with resveratrol. Following the predetermined incubation time, 20 μL of CCK-8 dye was added to each well, incubated for 3 hours at 37°C before measuring the absorbance at 450 nm.

### MAPK signaling assay for ERK expression by western blot analysis

These studies were conducted in whole cells lysates that were prepared from flasks containing 80–90% confluent cells following trypsinization. About 1–2 χ 10^6^ cells were plated per flask. The cells were allowed to attach and incubate overnight in serum free media. The cells were then treated with 100 μM resveratrol or 100 μM nicotine, washed with cold PBS and placed on ice. A RIPA buffer of 250 μL containing PMSF/protease III cocktail inhibitor was added to lyse the cells. The cells were then sonicated and kept on ice for 40 minutes. At the end of this period, the cell protein mixture was spun at 12000 rpm for 10 minutes, supernatant removed and kept on ice until used for assay. Protein concentration was determined using bovine serum-albumin as the standard, as described earlier^19^.

For western blot analysis, a total of 40 μg of cellular protein was loaded onto 12%SDS-polyacrylamide gels and electrophoresed for 1 h and 30 min, at a steady voltage of 120 V. The separated protein bands were then transferred to nitrocellulose membranes (Bio Rad Laboratories, Hercules, CA). The primary antibodies used for probing the nitrocellulose membrane overnight were obtained from Cell Signaling (Danvers, MA). The antibodies used were: anti-ERK1/2, anti-pERK1/2. Subsequently membranes were probed with horseradish peroxidase-conjugated secondary antibody (Pierce Biotechnology Inc., Rockford, IL). Enhanced chemiluminescence solution (ECL+, Amersham BioSciences, Piscataway, NJ) was used to visualize the bands. The band intensity was quantified using a STORM 860 Imager (Molecular Dynamics, Inc., Sunnyvale, CA).

### Confirmation of MAPK signals activation as measured by immunofluorescence imaging

These studies were conducted by plating 4 χ 104 cells per well in a 4-well Lab-Tek chamber slides (Becton Dickinson Labware, Franklin Lakes, NJ). The cells were attached for 24 hours in 10% FBS media before being transferred to serum free media overnight. The cells were then exposed to different treatments. For example: some cells were exposed to 100 μM resveratrol for 30 min or 100 μM nicotine for 3 min. Additional cells were pretreated with 100 μM resveratrol before being exposed to 100 μM nicotine. Control cells were not treated. After brief washing with cold PBS, cells from all of the above groups were fixed with 2% paraformaldehyde for 20 minutes at room temperature, permeabilized with 1% Triton X-100 in PBS for 5 minutes followed by extensive washing with PBS. Blocking was done using 1% bovine serum-albumin and 5% goat serum in PBS. Incubation of primary antibody to p-ERK (1:100 dil.) in 1% bovine serum-albumin, was continued for 24 hours at 4°C . Following incubation, the slides were washed 3 times, 10 minutes each with PBS. After washing, the cells were incubated with fluorescein isothiocyanate-conjugated anti-rabbit IgG antibody (1:50 dilution, Sigma, St. Louis, MO), at room temperature for 45 minutes. Slides were then washed extensively (3 times for 10 min) in PBS. Mounting media from Invitrogen Technologies (Carlsbad, CA) was used to mount the samples. The slides were then viewed under confocal microscope and images were taken using Fluorescent 2 software (Fluorescent Labsystems OY, Helsinki, Finland). The negative immunostaining controls were cells that were unexposed and incubated with secondary antibody.

### Measurement of cellular lipid peroxidation products induced by nicotine, with or without resveratrol pretreatment

Lipid peroxidation assay was conducted using MDA-586 method (Oxis Research, Portland, OR) on whole cell lysates. The cell lysates were obtained from cells treated with either 100 μM resveratrol (Sigma-Aldrich, St Louis, MO) for 30 min or 100 μM nicotine for 3 min, followed by a combination of both resveratrol and nicotine. Malondialdehyde (Sigma-Aldrich) was used as a standard. Both the standards and whole cell lysates were incubated for 1 h in a 45°C water bath with N-methyl-2-phenylindole (NM2P, dissolved in acetonitrile) and diluted with methanol together with concentrated HCl. The ratio of cell lysate to the volume of NM2P solution was 1:5.

### Statistical analysis

Experimental values were calculated as mean ± SEM of the number of experiments, as indicated in the legends. Data were evaluated for statistical significance with one-way ANOVA. A p-value of 0.05 or less was considered as statistically significant.

## RESULTS

### Effects of resveratrol and nicotine on cell proliferation of AR42J cells with or without resveratrol treatment

**Figure 1:**
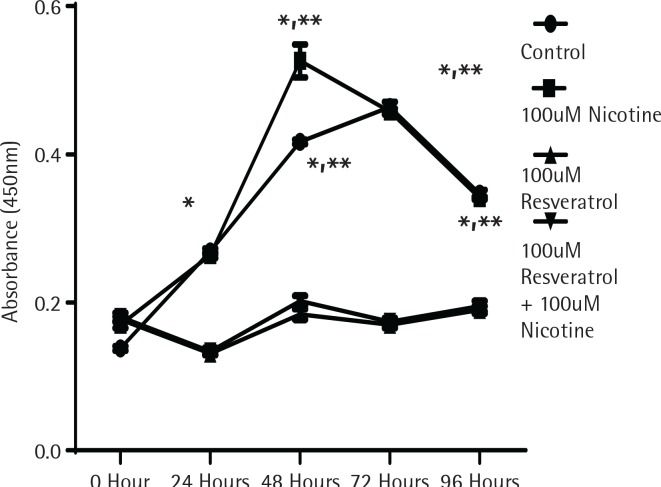
The effect of nicotine or resveratrol and nicotine on the proliferation of AR42J cells. The cells were plated in 96-well plates and allowed to attach overnight before transferring to 5% serum media for 10–12 hours before beginning of the study. The cells were then treated with 100 μM nicotine or 100 μM resveratrol and the proliferation measured at 24–96 h using a cell counting kit from Dojindo Molecular Technologies according to manufacturer’s instructions. The data points represent mean ± SEM of 5 experiments (* represents a significant difference from untreated or nicotine treated control; ** significant difference from treatments of both 100 μM Resveratrol and 100 μM Resveratrol + 100 μM Nicotine)

To determine whether the activation of MAPK signalling by nicotine provides direct influence on cell growth, cell proliferation experiments were performed using the MTT assay as described in the Methods section. Figure 1 shows the proliferation data measured by absorption spectrometry compared to control untreated cells designated by the incubation time in serum media. As shown in Figure 1, cells in control and nicotine treated groups showed a significant increase in cell proliferation after incubation for 48 h. The cells treated either with resveratrol alone or in combination with nicotine did not increase any cell proliferation, suggesting that resveratrol at the dose levels used provided a profound inhibitory effect to cell proliferation (p<0.05).

### Activation of ERK signal induction by nicotine was suppressed by resveratrol

**Figure 2:**
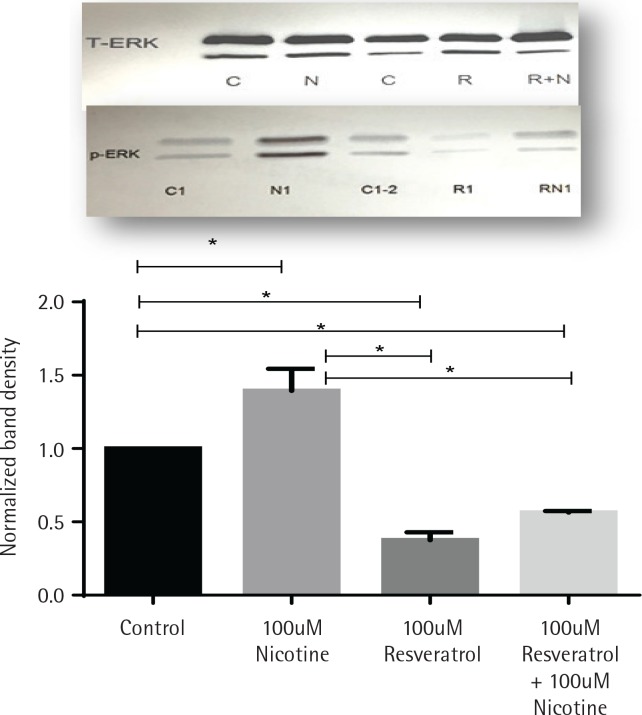
Induction of ERK1/2 in AR42J cells exposed to nicotine or resveratrol. Control untreated cells, cells exposed to 100 μM resveratrol for 30 min or 100 μM nicotine 3 min were lysed and the lysates were for western blotting. Upper panel: western blot visualization with ECL-plus using STORM Imaging software using T-ERK and p-ERK antibody. Lower panel: Band intensity showing the fold increase as mean ± SEM of 5 experiments; * referred to as significant between the two values; C, C1, C2 are control; N, N1, nicotine treated: R, R1, resveratrol treated; R+N, RN1, resveratrol + nicotine combined treatment.

The effects of nicotine on the activation of mitogenactivated protein kinase signal (ERK1/2, MAPK) in AR42J cells, treated with or without nicotine, were analysed by western blot analysis and are presented in Figure 2. AR42J cells were pretreated with 100 μM resveratrol for 30 min before being exposed to nicotine for 3 min. The induction of p-ERK1/2 signal was determined by western blot with primary p-ERK1/2 antibody. Figure 2 shows that ERK activation was induced by nicotine but not by resveratrol and it was significant when compared to both control and nicotine-treated cells (p<0.05). Treatment with resveratrol and nicotine increased the ERK activation, however there was no significant differences in p-ERK expression measured between the resveratrol-treated cells exposed either alone or in combination with nicotine.

### Confirmation of activated p-ERK1/2 on AR42J cells in response to resveratrol and nicotine treatment as measured by immunofluorescence

**Figure 3:**
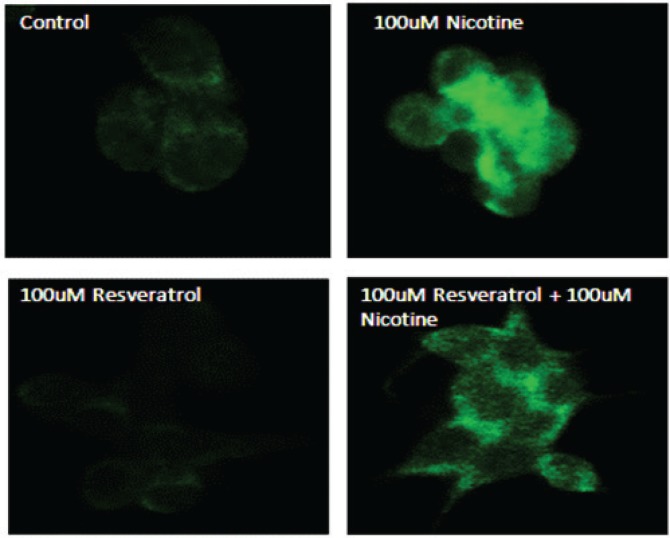
Induction of p-ERK1/2 in AR42J cells as indicated by immunohistochemistry. AR42J cells were grown and treated with 100 μM nicotine for 3 min; 100 μM resveratrol for 30 min. The cells were fixed with paraformaldehyde and treated with antibody to p-ERK1/2 for 1 h. After washing, the cells were treated with secondary antibody labeled with FITC. Slides were observed using a confocal microscope. Upper left panel: Control untreated cells probed with primary antibody to p-ERK1/2. Upper right panel: Cells exposed to nicotine for 3 min probed with primary antibody to p-ERK1/2. Lower left panel: Cells exposed to resveratrol for 30 min probed with primary antibody to p-ERK1/2. Lower right panel: Cells exposed to resveratrol for 30 min followed by nicotine for 3 min and probed with primary antibody to p-ERK1/2.

To further confirm the effects of resveratrol and nicotine on MAPK activation, we analysed the cells exposed to nicotine with or without resveratrol treatment with p-ERK antibody labeled with fluorescein by employing immunofluorescence imaging. Figure 3 shows the results of immunostaining data of control untreated cells and nicotine exposed cells that were compared to resveratrol-treated cells. As shown in Figure 3, the observed p-ERK1/2 signal is distributed throughout the cytoplasm for all groups tested. However, a considerably higher fluorescent intensity was observed for the nicotine-treated cells compared to control and resveratrol-treated cells, indicating the activation and distribution of increased p-ERK signal through the cytoplasm of these cells. The immunofluorescence staining in cells treated with a combination of resveratrol and nicotine was not different from that of resveratrol treatment alone, complementing the results shown in Figure 2.

### Effects of nicotine and resveratrol on MDA formation in AR42J cells

The effects of nicotine on formation of lipid peroxidation products in AR42J cells treated with or without resveratrol are presented in Table 1. Cells were treated with 100 μM nicotine for 3 min and 100 μM resveratrol for 30 min. For combination experiments, the cells were pretreated with 100 μM resveratrol for 30 min before being exposed to nicotine for 3 min. The results show that while the control untreated cells had an average MDA concentration of 0.04 μM/mg protein, MDA concentrations in the nicotine-treated conditions were increased significantly by 4.5-fold (an increase of 354%) from the control. With resveratrol treatment the MDA level was not significantly different from the control group. Compared to the nicotine-treated group, the MDA levels in this group were 50% lower (p<0.05). With combined treatment of resveratrol followed by nicotine, the MDA values were higher, however, these values were significantly lower than those of the nicotine group (a decrease of 35%).

**Table 1:** Lipid peroxidation as determined by MDA concentration in AR42J cells. Control untreated cells, cells exposed to 100 μM resveratrol for 30 min or with 100 μM nicotine 3 min were lysed after treatments and the lysates were used in an MDA-586 Assay (Oxis Research, Portland, OR). MDA levels were expressed as μM/mg protein. Results are mean ± SEM of 5 experiments.

*MDA (μM/mg protein) for 5 Experiments*			
	*Mean ± SEM*	*Mean ± SEM*	*Mean ± SEM*
Control	Nicotine	Resveratrol	Resveratrol Resveratrol + Nicotine
0.044 ± 0.0022	0.2 ± 0.0851	0.0996 ± 0.0188	0.13 ± 0.0271
	354% increase from control	50% decrease from nicotine	35 % decrease from nicotine
p-value	<0.05	<0.05	<0.05

## DISCUSSION

Resveratrol has been shown to suppress proliferation of a wide variety of tumor cells, including lymphoid and myeloid cancers, breast, colon, pancreas, stomach, prostate, head and neck, muscle, ovary, liver, lung and cervical cancers and melanoma^1,2,7,9,11,15,16^. This communication examines the mechanism of action of resveratrol on pancreas utilizing an established mutant pancreatic tumor cell line^21,22^ through nitric oxide (NO) signaling pathway. It is known that this signaling pathway contributed significantly to our understanding of pancreatic pathophysiology^23^. We have chosen this particular cell line for this study because of its stability and known characteristic property of retaining its physiological secretory status, as in primary acinar cells^22^.To establish and confirm the inhibitory effect of resveratrol, we have used nicotine to induce oxidative stress, and to determine whether or not its effects on these cells are regulated via activation of nitric oxide synthase pathway^21^.

The lipid peroxidation was measured in these cells after their treatment with nicotine in the presence or absence of resveratrol. As shown in Table 1, nicotine induces the generation of oxygen free radicals within the cell as measured by MDA. The data showed that nicotine induced robust increases in MDA formation in AR42J cells, compared to the control untreated cells, confirming nicotine-induced ROS formation. Treatment with resveratrol reduced MDA levels induced by nicotine, suggesting that resveratrol acts as an inhibitor in these cells through xanthine oxidase (XOD) pathway. Generation of oxygen radicals can occur through xanthine oxidase (XOD), which oxidizes hypoxanthine to xanthine liberating superoxide anion^24,25^. This pathway has been examined earlier in our laboratory^26^. It has been shown that the increase in ROS production is linked directly to oxidation of cellular macromolecules, which may cause direct cell injury or induce a variety of cellular responses through the generation of secondary metabolic reactive species^27^. Oxidative DNA damage can lead to genetic instability, as shown in studies with human lung cancer cell lines^28^. Our results show that resveratrol was able to reverse protein oxidation (Table 1), implicating its strong anti-oxidative effects.

Extensive research during the last two decades has suggested that, besides cardio-protective effects, resveratrol also exhibits anticancer actions. Mechanisms, by which resveratrol acts as an anti-carcinogen, affects cell signaling pathways, modulates the transcription factors, induces genes, regulates the enzyme activities and protein interactions, were examined earlier for in vitro and in vivo model systems, in a comprehensive review report^2^. Our study adds further support to anti-proliferative effects of resveratrol in pancreatic cancer cells (Figure 1).

The same concentration of nicotine that induced MDA formation (Table 1) also induced the activation of p-ERK1/2 signalling (Figure 2). As shown in Figure 2, cells exposed to 3 min of incubation with nicotine resulted in maximal induction of p-ERK1/2. These results are consistent with the data on p-ERK1/2 activation in cultured endothelial cells in which peak responses of p-ERK activation are shown to occur after 20 min of exposure followed by its return to baseline in 60 min^29^. Induction of p-ERK1/2 has also been observed in other mammalian cells exposed to H_2_O_2_^30,31^, where it was suggested that ERK can be activated by ROS. The induced activation of p-ERK signals by nicotine was eliminated by resveratrol when the cells were pretreated with resveratrol (Figure 2). These data suggest that induced MAPK signalling by nicotine was mediated via oxidative mechanisms and resveratrol acted as an inhibitor. In other studies allopurinol, an inhibitor of XOD system, has been shown to ameliorate the caerulein-induced pancreatitis^32^ and inhibition of XOD was shown to provide protection of organs against oxidative stress.

The activation of MAPK signals by nicotine and resveratrol was confirmed by increased immunofluorescence (Figure 3) showing distribution and co-localization of p-ERK1/2 within the cytosol. The induction of p-ERK1/2 in nicotine-treated cells was significantly higher than that of control untreated and resveratrol-treated cells. Resveratrol treatment reduced the fluorescence to control level, suggesting that p-ERK activation and co-localization of these signals in the cytosol may, in part, be mediated by oxidative stress signalling pathways.

Activation of pERK1/2 as a signal for MAPK pathway is considered indicative of growth, differentiation and development. Cell proliferation was significantly inhibited by resveratrol within the first 48 h when incubated in low serum media. In contrast, nicotine treatment induced a significant increase in proliferation (Figure 1). Resveratrol treatment significantly reduced the nicotine-induced cell proliferation, suggesting that oxidative species regulating p-ERK activation play an important role for induced cell growth by nicotine. It has been suggested that the induction of p-ERK1/2 by H_2_O_2_ is a cell-specific response^33^ where nicotine as an oxidant, may be able to utilize multiple pathways to produce mitogenic effects depending on the cell type. These findings suggest that in response to oxidative stress, activation of ERK signalling pathways plays a critical role in controlling cellular protection in the early stage. Recognition of these type of phenomena in endothelial cells^34^ explains the regulation of the MAP kinase signalling pathway by H_2_O_2_^33^, and also indirectly by nicotine as oxidant marker.

## CONCLUSIONS

In this study, we have examined exposure to resveratrol and nicotine induced effects on acinar cells that are mediated by pathways involving oxidative stress. Cell proliferation studies showed a significant difference in the effects between resveratrol and nicotine on AR42J cells. This indicates that, while nicotine exposure does result in the production of ROS within the cells, resveratrol can reverse these effects induced by nicotine. Thus, we can conclude that effects of resveratrol on pancreatic cell proliferation may be regulated, in part, by mechanisms involving oxidative stress. Because ROS has been shown to cause DNA single-strand breakdown^35^, it is reasonable to consider further investigation of the role of resveratrol in multiple signal transduction pathways, as well as its direct inhibitory effect in pancreatic cancer cell proliferation.

## CONFLICTS OF INTEREST

Authors have completed and submitted the ICMJE Form for Disclosure of Potential Conflicts of Interest and none was reported.
